# Targeting of anti-citrullinated protein/peptide antibodies in rheumatoid arthritis using peptides mimicking endogenously citrullinated fibrinogen antigens

**DOI:** 10.1186/s13075-015-0666-6

**Published:** 2015-06-10

**Authors:** Cátia Fernandes-Cerqueira, Elena Ossipova, Sunithi Gunasekera, Monika Hansson, Linda Mathsson, Anca I. Catrina, Yngve Sommarin, Lars Klareskog, Karin Lundberg, Johan Rönnelid, Ulf Göransson, Per-Johan Jakobsson

**Affiliations:** Rheumatology Unit, Department Medicine, Karolinska University Hospital, Solna, Rheumatology Clinic D2:01, 171 76 Stockholm, Sweden; Division of Pharmacognosy, Department of Medicinal Chemistry, Biomedical Centre, Uppsala University, Husargatan 3, 751 23 Uppsala, Sweden; Department of Immunology, Genetics and Pathology, Uppsala University, Dag Hammarskjölds v 20, 751 85 Uppsala, Sweden; Euro-Diagnostica AB, Lundavägen 151, 202 11 Malmö, Sweden

## Abstract

**Introduction:**

We have previously identified endogenously citrullinated peptides derived from fibrinogen in rheumatoid arthritis (RA) synovial tissues. In this study, we have investigated the auto-antigenicity of four of those citrullinated peptides, and explored their feasibility to target anti-citrullinated protein/peptide antibodies (ACPA).

**Methods:**

The autoantigenic potential of the fibrinogen peptides was investigated by screening 927 serum samples from the Epidemiological Investigation of RA (EIRA) cohort on a peptide microarray based on the ImmunoCAP ISAC® system. In order to assay for ACPA blocking, two independent pools of purified ACPA were incubated with the respective targeting peptide prior to binding to cyclic citrullinated peptide (CCP)2 using the CCPlus® ELISA kit.

**Results:**

Two peptides derived from the fibrinogen α chain, Arg573Cit (563-583) and Arg591Cit (580-600), referred to as Cit573 and Cit591, and two peptides from the fibrinogen β chain, Arg72Cit (62-81) and Arg74Cit (62-81) (Cit72 and Cit74), displayed 65 %, 15 %, 35 %, and 53 % of immune reactivity among CCP2-positive RA sera, respectively. In CCP2-negative RA sera, a positive reactivity was detected in 5 % (Cit573), 6 % (Cit591), 8 % (Cit72), and 4 % (Cit74). In the competition assay, Cit573 and Cit591 peptides reduced ACPA binding to CCP2 by a maximum of 84 % and 63 % respectively. An additive effect was observed when these peptides were combined. In contrast, Cit74 and Cit72 were less effective. Cyclization of the peptide structure containing Cit573 significantly increased the blocking efficiency.

**Conclusions:**

Here we demonstrate extensive autoantibody reactivity against *in vivo* citrullinated fibrinogen epitopes, and further show the potential use of these peptides for antagonizing ACPA.

## Introduction

Anti-citrullinated protein/peptide antibodies (ACPA) are a hallmark of rheumatoid arthritis (RA) and are present in 60 to 70 % of RA patients [1, 2]. ACPA are commonly detected by an enzyme-linked immunosorbent assay (ELISA), which employs either one or a number of synthetic cyclic citrullinated peptides (CCPs) [2]. ACPA are believed to emerge following immune responses against citrulline containing proteins, formed post-transcriptionally by deimination (known as citrullination) by means of specific peptidylarginine deiminases (PADs). Fibrinogen [3], α-enolase [4], vimentin [5], and collagen II [6] are well-characterized citrullinated proteins targeted by ACPA.

ACPA have been suggested to play a role in the pathogenesis of RA: the occurrence of these autoantibodies antedates the clinical onset by several years [7], they are associated with a more aggressive and destructive disease course (compared to the CCP-negative subset) [8], and it has been suggested that antibodies targeting citrullinated fibrinogen are involved in the development of arthritis in mice [9]. The molecular mechanisms behind the effects of ACPA have been addressed in several studies. Immune complexes formed by ACPA and citrullinated fibrinogen were able to co-stimulate human and murine macrophages via both Toll-like receptor 4 and FC gamma receptor pathways [10, 11]. It was also shown that anti-CCP antibodies could activate both the classical and the alternative complement pathways in dose-dependent manners *in vitro* [12]. In addition, the involvement of ACPA in bone metabolism was identified, giving evidence that anti-citrullinated vimentin antibodies cause osteoclastogenesis *in vitro* and *in vivo* in mice after intraperitoneal transfer of purified antibodies [13]. In line with these results, and adding to the concept of ACPA pathogenicity, ACPA levels were recently described to correlate with the increased presence of neutrophil extracellular traps (NETs) released during NETosis of both circulating and synovial fluid RA neutrophils, and RA NETs could be a source of citrullinated proteins [14].

Since ACPA are detected early in the time-course of the disease and are likely involved in the pathophysiology, one could speculate about the advantage of having a targeted therapy against ACPA. Such treatment might be possible by blocking ACPA with specific peptides, for example using peptides derived from citrullinated fibrinogen. In fact, a similar approach has been used for the blocking of autoantibodies against the cardiac β1-adrenergic receptor. A cyclic peptide (COR-1) that mimics the real epitope structure was shown to prevent autoantibody-mediated myocardial damage in an experimental model of immune cardiomyopathy [15, 16].

Fibrinogen is one of the most extensively characterized ACPA targets. We have previously identified endogenously citrullinated residues at positions 573 and 591 within the α fibrinogen chain, and at positions 72 and 74 in the β chain from human arthritic synovial tissues, using mass spectrometry (MS) [17]. Previously, several citrullinated and non-citrullinated fibrin-derived peptides from the α and β chains have been tested for recognition by ACPA [18]. A total of 18 citrullinated peptides out of 71 tested were found to contain epitopes recognized by RA CCP-positive sera. Also, circulating immune complexes containing citrullinated fibrinogen were shown to be present in plasma from CCP-positive RA patients [19].

In the current study, we have analyzed sera from RA patients for ACPA responses against peptides mimicking the endogenously citrullinated epitopes, in the form of citrullinated peptides generated *in vitro*, and also investigated if these epitopes could be employed to target purified anti-CCP2 immunoglobulin G (IgG) molecules.

## Methods

### Patients

Serum from 927 RA patients (newly diagnosed patients within 12 months of appearance of first symptoms; 402 CCP2 positive and 525 CCP2 negative) and 461 healthy controls from the Epidemiological Investigation of RA (EIRA) case-control cohort were collected. Control samples were randomly chosen from the Swedish population registry, matching for age, sex, and residential area. All serum and plasma samples were stored at −70 °C prior to analysis. All patients fulfilled the American College of Rheumatology-European League Against Rheumatism criteria for RA [20]. CCP2-positive RA individuals used for ACPA affinity purification were selected based on having high anti-CCP2 antibody levels (>300 AU/ml, Immunoscan CCPlus® assay, Euro-Diagnostica AB, Sweden). All patients gave written informed consent. This study was approved by the regional ethical committee at the Karolinska Institutet (96-174), and performed in accordance with the Declaration of Helsinki.

### Peptide identification and synthesis

Endogenously citrullinated fibrinogen peptides of the α chain, Arg573Cit (563-583) and the β chain, Arg72Cit (62-81) and Arg74Cit (62-81), in synovial tissue from RA patients were previously identified by MS [17]. α chain peptide Arg591Cit (580-600) was also identified in the study, but it was excluded from the results due to the stringent criteria used (the peptide contains N and Q). The occupancy rate for Cit591 was calculated to 0.8 %, using accurate mass-time tag and peak area for both *in vivo* citrullinated peptide and its *in vitro* citrullinated standard.

After optimization for solubility, length, and citrulline position, citrullinated and unmodified peptides were acquired with >95 % purity from ProImmune AB (Oxford, United Kingdom) and used in a peptide microarray. The corresponding N-terminally biotinylated peptides (ProImmune AB, purity >95 %) were used in the competition assays. The sequences of these peptides are illustrated in Table 1. Citrullinated or unmodified (that is, Arg containing) peptides Arg/Cit573, Arg/Cit72 and Arg/Cit74 were dissolved in sterile distilled water, whereas Arg/Cit591 were dissolved in 10 % dimethyl sulfoxide (DMSO). DMSO alone (10 %) did not show any reactivity in the assay. In addition, Arg/Cit573 peptides were synthesized in house using the OxymaPure/DIC method and no biotin was incorporated in the N-terminal end. Similar results were obtained when using either biotinylated or non-biotinylated Arg/Cit573 peptides (data not shown).

**Table 1:** Sequences of the fibrinogen α chain peptides 573 and 591 and β chain peptides 72 and 74

Peptide name	Peptide sequence
Arg573	HHP GIA EFP S**R**G KSS SYS KQF
Cit573	HHP GIA EFP S**X**G KSS SYS KQF
Arg591	SKQ FTS STS YN**R** GDS TFE SKS
Cit591	SKQ FTS STS YN**X** GDS TFE SKS
Arg72, 74	APP PIS GGG Y**R**A **R**PA KAA AT
Cit72	APP PIS GGG Y**X**A RPA KAA AT
Cit74	APP PIS GGG YRA **X**PA KAA AT

**R**, Arginine; **X**, Citrulline

Peptides derived from Cit573 (Table 2) were synthesized in house by either manual or microwave-assisted standard Fmoc-solid phase peptide synthesis (SPPS) on a 0.1 mM scale using HBTU/DIPEA (Sigma-Aldrich, St. Louis, MO, USA) as a coupling agent and piperidine (Sigma-Aldrich, St. Louis, MO, USA) as an Fmoc deprotecting agent. The two linear peptides (Cit573Lin1 and Cit573Lin2) were synthesized (C-terminal acid) on a NovasynTGT resin (Merck Millipore, Darmstadt, Germany) preloaded with Ser (0.2 mmol/g, Novabiochem, Merck Millipore, Darmstadt, Germany). Cit573Lin1 was obtained in 93 % crude yield, and MS analysis confirmed correct peptide identity: observed mass 1,573.65 (M + H)^+^, calculated mass 1,573.68 (M + H)^+^. The yield of Cit573Lin2 was 89 % with an observed mass of 1,153.43 (M + H)^+^ and calculated mass of 1,153.21 (M + H)^+^. Cit573 cyclic (Cit573Cyc) was synthesized on a Tentagel Rink Amide resin (0.18 mmol/g, Peptide International, KY, USA) using a C-terminal diaminobenzoic acid (Dbz) linker and an N-terminal cysteine for cyclization by native chemical ligation [21, 22]. The peptide-Dbz was acylated and activated to form the peptide N-benzylmidazolinone (Nbz) on the resin. Following the assembly of the peptide chain (peptide-Nbz), *in situ* thioesterification and cyclization was carried out in a pre-established cyclization/ligation buffer. Cit573Cyc was obtained in 46 % crude yield. MS analysis confirmed the peptide identify: experimental mass 2,433.93 ((M + H)^+^ deconvoluted from the doubly and triply ions), calculated mass 2,433.69 (M + H)^+^. Freeze-dried linear and cyclic peptides were purified by reversed phase high performance liquid chromatography (RP-HPLC; >95 % purity) as determined by analytical HPLC (UV 215 nm) and MS.

**Table 2:** Sequences of cyclic and truncated peptides based on the citrullinated fibrinogen α 573 peptide

Peptide name	Peptide sequence
Cit573Cyc	cyclo-CHHP GIA EFP S**X**G KSS SYS KQF
Cit573Lin1	GIA EFP S**X**G KSS SYS
Cit573Lin2	A EFP S**X**G KSS S

**X**, Citrulline; Cyc, cyclic; Lin1, Linear form 1; Lin2, Linear form 2

### Peptide microarray

Peptide microarray was performed as previously described [23]. Briefly, peptides (sequences in Table 1) were immobilized onto a chemically modified glass slide, sera from RA patients and healthy controls were applied into the reactions sites, and fluorescence intensity after incubation with anti-human IgG antibody was acquired in a laser scanner (LuxScan 10K, CapitalBio, Beijing, China). Final results for each citrullinated peptide were calculated by subtracting the intensity values of the corresponding arginine-containing control from the citrullinated peptide for all RA patients and controls. Cutoff values were determined as the 98th percentile of the healthy control responses. Microarray data are available in the ArrayExpress database under accession number [EMBL:E-MTAB-3606]*.*

### Preparation of human ACPA (anti-CCP2 IgG antibodies)

ACPA were affinity purified as recently described [24]. Two different ACPA pools were prepared for the competition assays: ACPA pool I, containing autoantibodies purified from 11 plasma or serum samples (plasma n = 4, sera n = 7); and ACPA pool II, containing 39 plasma or serum samples (plasma n = 38, serum n = 1). Before pooling, each purified ACPA sample was individually tested by in house ELISA for immunoreactivity to fibrinogen Cit573 and citrullinated fibrinogen 36-52 [24]. Anti-CCP2 reactivity was determined using the Immunoscan CCPlus® assay. All pooled samples were CCP2 positive.

### CCP2 ELISA and competition assays

ACPA pools I and II were used at a concentration of 1.2 μg/ml (8 nM IgG). The autoantibodies were incubated with the fibrinogen and fibrinogen-derived peptides at room temperature for one hour. The mole quantities of peptides tested were: 0.016, 0.16, 1.6, 16, 22, 49, 60, 80, 120, 160, 216, and 320 nmol in an incubation volume of 225 μl. ACPA blocking by the fibrinogen peptides was subsequently assessed by measuring the remaining anti-CCP2 reactivity. Cit573 and Cit591 were equally combined and co-tested for ACPA blocking. Arginine peptides (573, 591, 72, and 74) were used as negative controls in all assays.

To further explore the blocking potential of Cit573, a retrieval assay was performed, that is, the ability of the peptide to compete with already CCP2-bound ACPA was tested. ACPA pool I was added to the CCP2 plate and incubated for one hour at room temperature, followed by the addition of the peptides (citrulline and unmodified peptides) at mole quantities ranging from 16 to 320 nmol. ACPA pools incubated with no peptide were defined as positive control, giving the maximum values of anti-CCP2 reactivity. Arbitrary units were calculated according to the standard curve given by the ELISA kit. Each experiment had one standard curve. Mean values were calculated and results are expressed as a function of percentage of inhibition of ACPA from the peptide amount (nmol).

### Statistical analysis

Results are presented as mean ± standard error (SEM) of values (two to seven experiments per ACPA pool), and were compared using the Student’s *t*-test for unpaired comparisons using GraphPad Prism 6 software (La Jolla, CA, USA). Median values were also calculated in order to consider non-Gaussian distributions. As the mean and median values were generally coincident, the Mann-Whitney *U* test was not selected for statistical analysis. *P* <0.05 was assumed to denote a significant difference. Relative IC_50_ values (IC_50_ definition according to International Union of Pharmacology Committee on Receptor Nomenclature and Drug Classification – ‘the molar concentration of an antagonist that reduces the response to an agonist by 50 %’ [25]) were also calculated using GraphPad Prism 6 software. Cutoffs for the ISAC system chip data were calculated as the 98th percentile reactivity among the 461 EIRA controls investigated in parallel to the 927 RA patients.

## Results

### Peptide microarray - immunogenicity of fibrinogen peptides

**Fig. 1:**
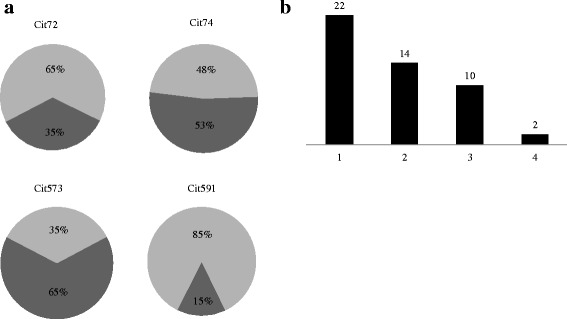
**a**. Percentage of responses among tested patients. Immune reactivity (%) against the respective peptide in sera from CCP2-positive patients. Positive immune reactivity (%) (dark grey) against each individual citrullinated fibrinogen peptide in sera from CCP2-positive patients; light gray represents negative immune response (%) against each individual citrullinated peptide in CCP2-positive sera tested. **b**. Relative frequency (%) of patient sera reacting to one, two, three, or four citrullinated fibrinogen peptides

Immune reactivity of RA (n = 927) and healthy control (n = 461) sera against four citrullinated fibrinogen peptides was investigated. When all analyses were aligned to the same 98 % specificity level, the diagnostic sensitivity among all 927 RA patients was 20.0 % for Cit72, 25.2 % for Cit74, 31.5 % for Cit573, and 9.8 % for Cit591. When the patients were dichotomized according to anti-CCP2 status, the corresponding figures were 35.1 % (Cit72), 52.5 % (Cit74), 65.4 % (Cit573), and 14.7 % (Cit591) for the anti-CCP2-positive patients, (Fig. 1a) and 8.4 % (Cit72), 4.4 % (Cit74), 5.5 % (Cit573), and 6.1 % (Cit591) for the anti-CCP2-negative patients (data not shown). Only a subgroup of the controls investigated with anti-CCP2 with the ISAC chip had anti-CCP2 data. However, in a more recent and larger evaluation of EIRA where 578 controls were investigated for anti-CCP2, nine of these controls were found to be anti-CCP2 positive. The corresponding diagnostic specificity for anti-CCP2 is thus 98.4 %, closely resembling the chosen ISAC specificity of 98 %. Figure 1b demonstrates the proportion of all patient sera (both CCP2 positive and negative) that were positive for up to four of these peptides. A total of 47 % of the 927 patients tested showed positive antibody response to at least one of the four citrullinated fibrinogen peptides. When stratified, 22 % showed reactivity against one of the four peptides, 14 % showed reactivity against two peptides, 10 % showed reactivity against three peptides, and 2 % showed antibody responses to all four citrullinated fibrinogen peptides (Fig. 1b). Cutoff values were applied as the 98th percentile of the healthy control responses; 2 % of control population showed weak positive response to each peptide tested.

### ACPA blocking - inhibition of reactivity in the anti-CCP2 ELISA

We next sought to investigate if fibrinogen-derived peptides (citrullinated and unmodified versions) were able to compete with CCP2 for binding of patient-derived ACPA. Hence we performed an ‘ACPA competition’ experiment, where purified human ACPA was pre-incubated with our fibrinogen peptides before being assayed on the anti-CCP2 ELISA.

**Fig. 2:**
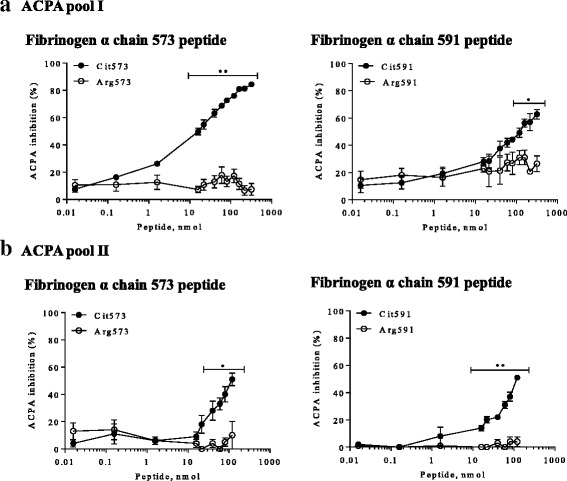
Dose-response curves representing the percentage of ACPA targeted by fibrinogen α chain peptides. ACPA was incubated with the respective peptides at different concentrations for one hour at room temperature, before proceeding with the anti-CCP2 ELISA. **a**. ACPA (pool I) blocking with citrulline or arginine-containing fibrinogen peptides from the α chain, 573 (left panel) and 591 (right panel). **b**. ACPA (pool II) blocking with citrulline or arginine-containing fibrinogen peptides from the α chain, 573 (left panel) and 591 (right panel). X-axes show peptide amount (nmol). Y-axes show ACPA inhibition levels (%). Circles represent means of two to seven experiments per ACPA pool and error bars represent SEM. ^*^ *P* <0.05, ^**^ *P* <0.001

When incubated with ACPA pool I, soluble linear Cit573 resulted in the highest degree of ACPA blocking, achieving 84 % inhibition of reactivity, with an IC_50_ of 59 μM ± 8 (n = 6; Fig. 2a, left panel). Fibrinogen linear peptide Cit591 resulted in a similar dose-response curve as Cit573. A maximum of 63 % ACPA blocking was recorded for Cit591, and the IC_50_ calculated was 194 μM ± 3 (n = 2; Fig. 2a, right panel).

When the two peptides Cit573 and Cit591 were equally mixed and tested in the competition assay, a significantly higher degree of ACPA blocking was achieved, reaching 91 % inhibition (data not shown). Using ACPA pool II, Cit573 resulted in a slightly weaker ACPA inhibition in comparison to that observed with ACPA pool I (Fig. 2b, left panel). Approximately 50 % of ACPA in the anti-CCP2 plate were blocked (IC_50_ 548 μM ± 100, n = 4). On the other hand, ACPA pool II targeted by Cit591 showed a similar result to that observed with ACPA pool I (Fig. 2b, right panel; IC_50_ 412 μM ± 146, n = 4). When equally combined, Cit573 and Cit591 blocked up to 75 % of ACPA from pool II (data not shown).

No major inhibition was observed for any of the two ACPA pools, when using the unmodified peptide version Arg573 in the same experimental conditions as the citrullinated fibrinogen peptides (Fig. 2a and b, left panels). On the other hand, Arg591 displayed 30 % inhibition when incubated with ACPA pool I (Fig. 2a, right panel), but no inhibition was detected when incubated with ACPA pool II (Fig. 2b, right panel). In comparison, a maximum ACPA inhibition of 35 % and 26 % was observed for the citrullinated fibrinogen β chain peptides Cit72 and Cit74, respectively, while no inhibition was registered with the corresponding unmodified peptides (data not shown). Retrieval experiments were performed using the Cit573 peptide in order to evaluate the capability of this peptide to compete with CCP2 for already bound ACPA. Addition of Cit573 reverted up to 80 % of ACPA binding to the CCP2 ELISA plate (data not shown). No relevant retrieval capacity was observed for the arginine control peptide.

**Fig. 3:**
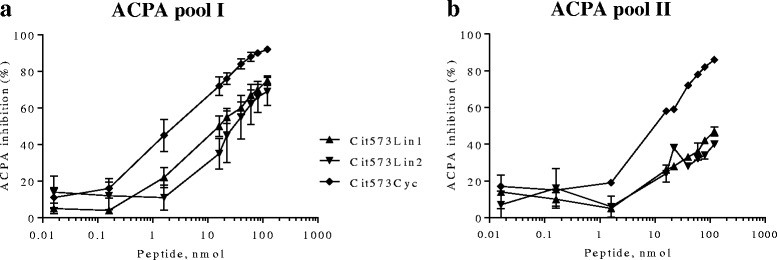
Dose-response curves representing the percentage of ACPA targeted by fibrinogen 573-derived peptides. ACPA was incubated at different concentrations of the respective peptide for one hour at room temperature before proceeding with the anti-CCP2 ELISA. **a**. ACPA (pool I) blocking with the Cit573 peptide, with the Cit573 truncated or cyclic forms or with the unmodified arginine-containing 573 peptide as control. **b**. Corresponding experiment as in **(a)** but using ACPA pool II. X-axes show peptide amount (nmol). Y-axes show ACPA inhibition percentage levels (%). Circles represent means of two to seven experiments per ACPA pool

To determine whether cyclization of the peptides improves ACPA targeting, a cyclic version of the full-length Cit573 peptide was synthesized (Cit573Cyc) and tested in the ACPA competition assay (Fig. 3). The cyclic version of Cit573 reached 92 % of ACPA inhibition (Fig. 3a), with an IC_50_ of 28 μM ± 5 (n = 3) when incubated with ACPA pool I. The full residue span within Cit573 may not be necessary for optimal ACPA binding, therefore two truncated linear peptides of 15 and 11 residues (Cit573Lin1 and Cit573Lin2), originating from the full-length Cit573, were tested and found to provide 75 % (IC_50_ 51 μM ± 6, n = 3) and 69 % (IC_50_ 123 μM ± 18, n = 2) ACPA inhibition, respectively (Fig. 3a).

The linear truncated peptides were also incubated with ACPA pool II. Here, Cit573Lin1 accomplished 47 % inhibition, with an IC_50_ of 186 μM ± 67 (n = 2), while Cit573Lin2 achieved 40 % inhibition, with an IC_50_ of 105 μM ± 34 (n = 2) (Fig. 3b). Interestingly, the cyclic form of the Cit573 peptide performed similarly in ACPA pool I (approximately 90 %) as in ACPA pool II (Fig. 3b).

## Discussion

In the present study we demonstrated extensive autoantibody reactivity against citrullinated fibrinogen epitopes, previously identified *in vivo* in RA synovial membranes [17]. We also showed that citrullinated peptides, corresponding to these *in vivo* modified epitopes, can be used as targeting agents, blocking a significant portion of ACPA binding to CCP2, forming the basis of a possible future therapeutic approach for ACPA-positive RA.

In the current study we have analyzed the antibody response towards four citrullinated fibrinogen peptides in sera from 927 RA patients, and 47 % of the patients were positive for antibodies against at least one of the peptides. The majority of antibody-positive patients were identified in the CCP2-positive subset, but interestingly, between 4 and 8 % of CCP2-negative patients also displayed reactivity against the citrullinated fibrinogen peptides, supporting previous findings that the CCP2 detection kit leaves out patients who produce antibodies against citrullinated proteins [26, 27].

In addition to the results described here, the same citrullinated fibrinogen peptides were also tested in a pre-RA cohort [28]. In that study, it was shown that antibodies against Cit573 and Cit74 were detected at low frequencies before RA onset, but at significantly increased frequencies after disease onset, suggesting an involvement in RA progression. Antibodies against Cit591 and Cit72, on the other hand, were detected at the earliest time points, pointing to a role in disease onset.

It has been suggested that ACPA mediate disease by promoting osteoclastogenesis [13], activation of the complement system [12], induction of TNF-α secretion [10, 11], and enhancement of NETosis [14]. In mice, ACPA targeting fibrinogen has been shown to enhance development of arthritis [9]. Since we know that fibrinogen is citrullinated *in vivo*, and that the specific epitopes investigated here are expressed *in vivo* [17], we hypothesize that ACPA of these specificities will be involved in RA pathogenesis. Hence, we suggest that these specific peptide sequences could be used in the development of novel drugs that will target pathogenic ACPA [29].

Additionally, and taking into account the possible association of certain citrullinated peptides (Cit591 and Cit72) with the disease onset, an alternative therapy would be to induce tolerance of Cit591- or Cit72-reactive CCP-positive RA patients with a Cit591- or Cit72-derived compound. The use of self-antigens (or closely-related structures) to induce tolerizing effects on dendritic cells has, for instance, been previously used in cancer. Farkas *et al*. demonstrated that mucin 1 transgenic mice developed transient tolerization of splenic dendritic cells when intravenously immunized with a mucin 1 peptide [30, 31]. In systemic lupus erythematosus (SLE), antigen-induced tolerance has been widely addressed. A nucleosomal histone epitope (H4_71-94_) was shown to delay lupus nephritis when administered (subcutaneously) to lupus-prone mice [32, 33], and it was also shown to suppress the disease in mice through nasal tolerance [34]. In addition, mice with experimental SLE were shown to have improved when treated with a peptide with a structure based on a complementary determining region 1 of a human anti-DNA autoantibody [35].

Competition with circulating autoantibodies has been successfully described in an animal model of immune cardiomyopathy. In this model, autoantibodies recognizing the β1-adrenergic receptor mediate myocardial damage [36]. The pathogenic autoantibodies were targeted using cyclic peptide COR-1, which mimics the main epitope of β1-adrenergic receptor, and this approach prevented myocardial damage. The safe usage of COR-1 has since been demonstrated in a phase I clinical trial [16]. Moreover, the blocking capacity of aptamers (small nucleic acids) was successfully evaluated in neonatal rat cardiomyocytes treated *in vitro* with anti-β1-adrenergic receptor autoantibodies [37]. Also in models of SLE, where autoantibodies targeting double-stranded DNA have been reported to cause glomerulonephritis [38], administration of soluble peptides that bind double-stranded DNA have been shown to protect against antibody-mediated experimental renal injury [39]. One relevant fact to discuss is if peptides or small molecules carrying an epitope may give rise to harmful immune complexes. Considering the size of the peptides (for example, approximately 2 kDa), an immune reaction against those is unlikely. In support, Diamond *et al*. in 2011 [40] showed that the administration of a soluble peptide (peptide with a consensus sequence that interacts with anti-dsDNA antibodies, and inhibits DNA binding and cross-reacts with anti-N-methyl-D-aspartate receptor antibodies) to mice previously injected in the hippocampus with a monoclonal anti-dsDNA antibody (R4A), did not induce aggregation or formation of immune complexes, and the combination was not toxic. On the contrary, the soluble peptide prevented antibody-mediated tissue damage by restricting the deposition of the antibodies in the glomeruli.

In line with these ideas, we have explored the capacity of peptides mimicking the endogenously citrullinated fibrinogen epitopes to prevent purified human ACPA from binding to CCP2. We have demonstrated the capacity of citrullinated fibrinogen peptides (Cit573 and Cit591) to target affinity purified human ACPA. When combined, the peptides blocked up to 91 % of the antibodies. Cyclization of the Cit573 peptide improved ACPA blocking, suggesting a more stable epitope presentation, and activity was retained in truncated versions of Cit573, demonstrating the possibility of simplifying syntheses and production of blocking peptides. In order to target the majority of ACPA, a large excess of blocking peptide was required. However, a rather small fraction of all ACPA sub-specificities [24] is likely to be subject to blocking when using only one single specificity, for example Cit573, at a low concentration.

B-cell depletion therapy (rituximab) has been shown to be more efficient in CCP-positive RA patients, in comparison to the CCP-negative subset [41–43]. The depletion of ACPA-producing CD20^+^ B cells correlates with improved disease activity, although re-population of naïve B cells induces relapse [44]. Hence, it would be an interesting approach to target the ACPA directly, hampering their capacity to propagate inflammation and induce tissue damage. We argue that blocking of ACPA with molecules based on the structures of citrullinated fibrinogen peptides might be used for such a purpose, and our data provides proof of the principle that blocking of ACPA may be developed into a new means of treating RA.

## Conclusions

We demonstrate that *in vivo* citrullinated fibrinogen epitopes found in RA synovial tissue are auto-antigenic. These peptides can be used as additional biomarkers for studies of ACPA sub-specificity profiles, as recently reported [28]. We also demonstrate that these citrullinated peptides can be used as probes for development of ACPA-blocking compounds preventing, for instance, the osteoclastogenesis and bone loss induced by ACPA or other ACPA-pathogenic effects [13].

## Abbreviations

ACPA: Anti-citrullinated protein/peptide antibodies
CCP2: Cyclic citrullinated peptide 2
Cyc: Cyclic
Dbz: C-terminal diaminobenzoic acid
DMSO: Dimethyl sulfoxide
EIRA: Epidemiological Investigation of rheumatoid arthritis
ELISA: Enzyme-linked immunosorbent assay
HPLC: High performance liquid chromatography
Lin: Linear
MS: Mass spectrometry
Nbz: N-benzylmidazolinone
NETs: Neutrophil extracellular traps
PADs: Peptidylarginine deiminases
RA: Rheumatoid arthritis
SEM: Standard error
